# *MYCN*-amplified stage 2/3 neuroblastoma: excellent survival in the era of anti-G_D2_ immunotherapy

**DOI:** 10.18632/oncotarget.20513

**Published:** 2017-08-24

**Authors:** Brian H. Kushner, Michael P. LaQuaglia, Shakeel Modak, Suzanne L. Wolden, Ellen M. Basu, Stephen S. Roberts, Kim Kramer, Karima Yataghene, Irene Y. Cheung, Nai-Kong V Cheung

**Affiliations:** ^1^ Department of Pediatrics, Memorial Sloan Kettering Cancer Center, New York, NY 10065, USA; ^2^ Department of Radiation Oncology, Memorial Sloan Kettering Cancer Center, New York, NY 10065, USA

**Keywords:** neuroblastoma, anti-G_D2_ antibody, autologous transplantation, cytokine, MYCN amplification

## Abstract

High-risk neuroblastoma (HR-NB) includes *MYCN*-amplified stage 2/3, but reports covering anti-G_D2_ immunotherapy, which recently became standard for HR-NB, do not provide details on this subset. We now report on all 20 *MYCN*-amplified stage 2/3 patients who received induction chemotherapy at our center during the era of consolidation with anti-G_D2_ antibody 3F8/ granulocyte-macrophage colony-stimulating factor (GM-CSF) (2000-2015). Early in this period, consolidation included autologous stem-cell transplantation (ASCT). Event-free survival (EFS) and overall survival (OS) were estimated using Kaplan-Meier analyses.

With induction, 19/20 (95%) patients achieved complete/very good partial remission (CR/VGPR) but one had progressive disease with early death. One responder did not receive consolidation and died of relapse. Five-year post-diagnosis EFS/OS rates for all 20 patients were 72%/84%. The 18 CR/VGPR patients who received consolidation had EFS/OS 81%/94% at five years from starting 3F8/GM-CSF: 4/4 ASCT patients remained relapse-free, while 11/14 non-ASCT patients remained relapse-free and two of the three relapsed patients achieved 2^nd^ CR (consolidated by retreatment with 3F8/GM-CSF) and remained in 2^nd^ CR at 36+ and 95+ months post-relapse. The 14 non-ASCT patients had EFS/OS 73.5%/93% at five years from starting 3F8/GM-CSF.

This subset appears to have a good prognosis with contemporary multi-modality therapy, possibly even without ASCT.

## INTRODUCTION

High-risk neuroblastoma (HR-NB) has long included *MYCN*-amplified stage 2 or 3 disease [1]. Because this entity is found in <10% of NB patients [1], the literature on this subset is limited [2–15], with the largest reported group-wide studies including 12-to-32 patients [4–6, 8–14]. Only one report covers this subset exclusively [9]. Other reports use definitions of *MYCN* amplification that are no longer accepted (*MYCN* copy number ≥3 [8, 10, 12] or various threshold values [13]). Analysis is further hampered because several reports, [4–8, 10–12, 16, 17] in presenting outcome data, group these patients with *MYCN*-amplified stage 1, *MYCN*-amplified stage 4S, and/or *MYCN*-non-amplified stage 3 (which we reported has an excellent outcome when treated by surgery alone at diagnosis [18]). Further, therapy for low-stage *MYCN*-amplified NB has varied widely from surgery alone [13] to myeloablative therapy with autologous stem-cell transplantation (ASCT) [9, 11, 13, 16]; details of treatment were not provided in the largest cohort of stage 3 reported to date [15].

With conventional chemotherapy alone, *MYCN*-amplified stage 2/3 patients typically relapsed in the primary as well as in distant sites [2–4, 7–9, 14]. ASCT plus local radiation therapy (RT) improved outcome in a small series (n=12), [9] but ASCT did not improve event-free survival (EFS) or overall survival (OS) of either *MYCN*-amplified stage 2 patients (n=39) collected by the International NB Risk Group (INRG) [13] or stage 3 patients (n=72) deemed high risk (including *MYCN*-non-amplified disease) in a national study [14]. Other reports concerning ASCT ± RT do not provide outcome data on *MYCN*-amplified stage 2/3 [11, 16].

Anti-G_D2_ antibodies such as dinutuximab and 3F8 are active against HR-NB [19]. Although no study compares efficacy, it is reasonable to assume approximately comparable anti-NB activity of different anti-G_D2_ antibodies. Immunotherapy using these agents recently became standard for HR-NB. However, reports showing a benefit do not include [20], or do not provide details on [21], stage 2/3 patients. We reported on stage 4 HR-NB treated at Memorial Sloan Kettering (MSK) with 3F8 alone [22] or plus granulocyte-macrophage colony-stimulating factor (GM-CSF) [23]. We now present results with *MYCN*-amplified stage 2/3.

Since 1990, the MSK treatment program for HR-NB has included dose-intensive chemotherapy [24, 25] and tumor resection [26] for induction, followed by consolidation using 3F8 [23] and local RT [27]. We added ASCT and isotretinoin for consolidation in 2000 after those treatments were reported as beneficial, [28] but we discontinued ASCT (though not isotretinoin) in 2003 because published ASCT studies [29–31] showed no survival advantage compared to the earlier MSK non-ASCT programs that used 3F8 without cytokines [22, 23]. We found no significant difference with or without ASCT in EFS or OS of stage 4 HR-NB patients in CR consolidated with 3F8/GM-CSF+isotretinoin plus local RT [23, 32]. We now report excellent outcome of *MYCN*-amplified stage 2/3, with or without ASCT.

## RESULTS

### Patient characteristics

The 20 patients (male:female, 11:9) had features typical of *MYCN*-amplified stage 2 (n=2) or stage 3 (n=18) HR-NB, including young age (10-75 [median 25] months), abdominal site, predominance of unfavorable histology, and elevated serum levels of lactate dehydrogenase at diagnosis (Table 1).

**Table 1:** Clinical and biological features

Patient #/sex/age at Dx/stage/site	LDH (U/L)	Ferritin (ng/mL)	ALK	Histology	Pathology at 2^nd^-look surgery	Outcome (time from Dx)
A. Patients without consolidative therapy						
*Complete remission with induction but no subsequent consolidation*						
1/M/32m/3/RP	5300	74	F1174L	…	(-)	PD (abd, bones) at 18m. Dod at 36m
*PD with induction*						
2/M/19m/3/R Adrenal	…	169	Wild type	UH	(+)	PD (abd) at 5m. Dod at 6.5m
B. Non-ASCT patients with 1^st^ remission consolidated by 3F8/GM-CSF, radiotherapy, and isotretinoin						
*Patients in continuing CR*						
3/F/10m/3/L Adrenal	3958	197	…	UH	(+)	CR at 162m
4/M/54m/3/RP	1637	111	Wild type	UH	(-)	CR at 100m
5/M/25m/3/L Adrenal	1510	185	Wild type	UH	(+)	CR at 99m
6/F/37m/2/L Adrenal	2789	52	…	UH	…	CR at 92m
7/M/18m/3/L Adrenal	2051	175	Wild type	FH	(+)	CR at 84m
8/F/59m/3/L Adrenal	1953	461	Wild type	UH	(+)	CR at 61m
9/M/48m/2/L Adrenal	401	…	Wild type	UH	(+)	CR at 43m
10/F/25m/3/RP	2102	…	R1275L	UH	(-)	CR at 34m^a^
11/M/14m/3/R Adrenal	968	…	Wild type	UH	(+)	CR at 28m^b^
12/M/75m/3/R Adrenal	4955	…	Wild type	UH	(+)	CR at 26m^b^
13/M/15m/3/R Adrenal	2619	166	Wild type	UH	(+)	CR at 24m^b^
*Patients who relapsed*						
14/M/31m/3/L Adrenal	2000	…	Wild Type	UH	(-)	PD (pelvis) at 59m. 2^nd^ CR 94m from relapse
15/F/47m/3/L Adrenal	624	…	F1174L	UH	(-)	PD (thorax) at 10m. 2^nd^ CR 34m from relapse
16/M/13m/3/L Adrenal	872	448	Wild Type	FH	(+)	PD (abd) at 10m. Dod at 13m
C. ASCT patients with 1^st^ remission consolidated by 3F8/GM-CSF, radiotherapy, and isotretinoin						
17/F/51m/3/RP	600	…	…	UH	(+)	CR at 206m
18/F/22m/3/L Adrenal	8189	…	…	…	(+)	CR at 202m
19/F/18m/3/RP	2642	125	…	FH	(+)	CR at 195m
20/F/19m/3/RP	613	154	Wild type	UH	(+)	CR at 145m

abd, abdomen; ASCT, autologous stem-cell transplantation; Dod, died of disease; Dx, diagnosis; F, female; FH, favorable histology; LDH, lactate dehydrogenase; M, male; PD, progressive disease; RP, retroperitoneal; UH, unfavorable histology.

^a^ Started difluoromethylornithine (DFMO)^33^ after completing 3F8/GM-CSF+isotretinoin.

^b^ Started vaccine^34^ after completing 3F8/GM-CSF+isotretinoin.

### Overview of outcome of the entire cohort

**Figure 1:**
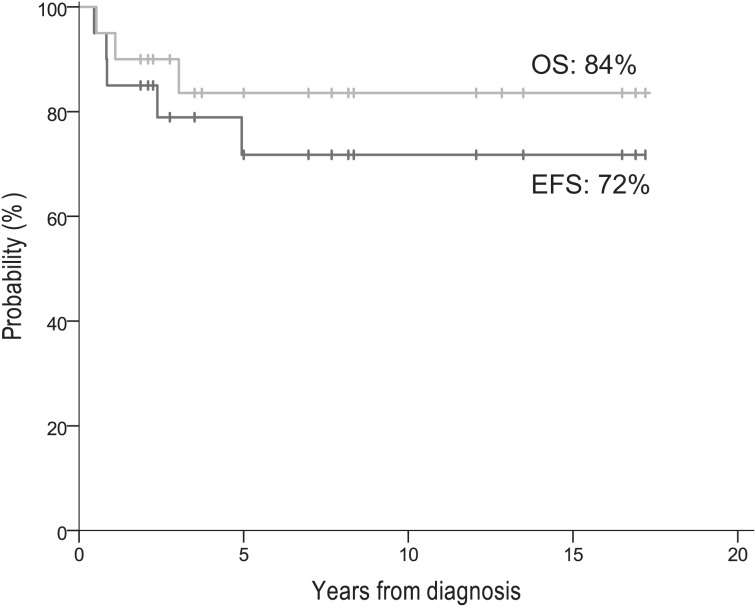
EFS and OS of all 20 patients

Of the 20 patients (Table 1), 19 (95%) achieved CR/VGPR with induction while one patient (#2) had PD with induction and died of PD at 6.5 months post-diagnosis. One CR patient (#1) received no consolidation (social reasons), had a widespread relapse documented at 18 months, and, despite aggressive salvage therapy, died of PD at 36 months post-diagnosis. The other 18 CR/VGPR patients received consolidation with 3F8/GM-CSF+isotretinoin+RT, including four with and 14 without prior ASCT. Minimal residual disease (MRD) was negative before 3F8/GM-CSF in 17 patients and positive in only one patient (#20), and was negative in all 18 patients after 2 cycles of this immunotherapy. The number of patients with either favorable histology (n=3) or an ALK mutation (n=3) was too small to assess for possible prognostic significance (Table 1). At 5 years from diagnosis, the entire cohort of 20 patients had EFS 72% ± standard error 11% and OS was 84% ± 9% (Figure 1).

Five patients had pathologic CR at 2^nd^-look surgery (Table 1) including the sole CR patient (#1) who did not receive consolidation and eventually relapsed (see above), and four non-ASCT patients, two of whom relapsed (patients #14 and #15). With thoraco-abdominal explorations, gross total resections were achieved at 2^nd^-look surgery in all but one patient (#20; ~80% resected, to avoid injury to portal vein and pancreatic ducts). Surgical complications included nephrectomy (patient #5), cholecystectomy for gall bladder obstruction (patient #13), hypertension from renal vasculature insufficiency (patient #14), and recurrent pleural effusions (patient #18). No unexpected chemotherapy-related complications occurred.

### Outcome of patients consolidated with 3F8/GM-CSF+isotretinoin

**Figure 2:**
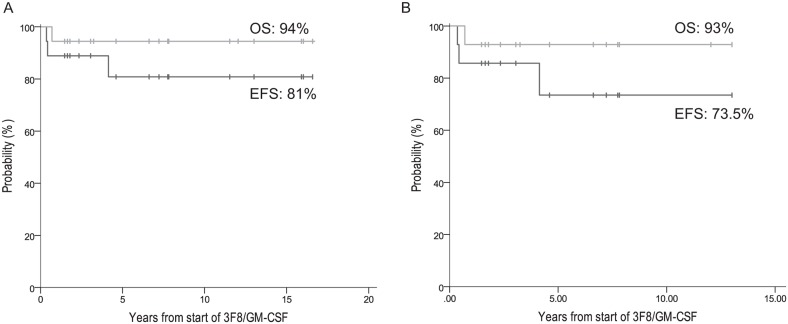
Patients in complete remission treated with 3F8/GM-CSF+isotretinoin EFS and OS of: **(A)** all 18 patients, and **(B)** the 14 non-ASCT patients.

At five years from the start of 3F8/GM-CSF, the EFS and OS rates of the 18 patients whose CR was consolidated with 3F8/GM-CSF+isotretinoin+RT were, respectively, 81% ± 10% and 94% ± 5% (Figure 2A). All four ASCT patients remain in CR at 145-206 (median 199) months from diagnosis, including the only patient (#20) with MRD detected in bone marrow (BM) pre-3F8/GM-CSF (MRD was negative when re-assessed after cycle 2 of 3F8/GM-CSF).

Eleven of the 14 non-ASCT patients remain relapse-free at 24-162 (median 60) months from diagnosis and 19-156 (median 55) months from the start of 3F8/GM-CSF, including four who received additional consolidative adjuvant (experimental and unproven) therapy with DFMO [33] (n=1) or a vaccine [34] (n=3). Two of the three patients who relapsed – one early at 10 months and one late at 59 months from diagnosis, both only in soft tissue outside the RT fields – have achieved prolonged ongoing continuous 2^nd^ CRs (95+ months for patient #14, 36+ months for patient #15). Their successful salvage therapy included previously described 2^nd^-line chemotherapy regimens, [35–37] resection of relapsed tumor, and local RT (21 cGy [26]). They also received repeat treatment with 3F8/GM-CSF+isotretinoin to consolidate their 2^nd^ CRs, as described [38], plus additional immunotherapy using a vaccine [34]. The other relapse was early, at 10 months, in soft tissue within the RT field, followed three months later by death from PD (patient #16). Overall, this cohort of 14 non-ASCT patients had EFS 73.5% ± 14% and OS 93% ± 7% at 5 years from the start of 3F8/GM-CSF (Figure 2).

As previously noted [23], 3F8/GM-CSF had manageable toxicities – hence, treatment was outpatient for the non-ASCT and the ASCT patients. 3F8 caused grade 1-2 generalized pain and urticaria but no unexpected toxicities [23, 32]. The only grade 3 toxicity of 3F8/GM-CSF was transient hypertension in the 1^st^ cycle of one patient (#13). Common side-effects of isotretinoin were grade 1-2 dry skin and cheilitis.

## DISCUSSION

This report is the first to focus on *MYCN*-amplified localized NB in the era that began when anti-G_D2_ immunotherapy became standard of care for HR-NB. This period coincided with the routine use of dose-intensive induction and consolidation with local RT and isotretinoin for HR-NB. Regarding *MYCN*-amplified stage 2/3, our experience shows that 1) dose-intensive chemotherapy and surgery can achieve a high CR rate, and 2) excellent long-term survival is associated with consolidation using anti-G_D2_ antibody, isotretinoin, and RT. The 5-year EFS/OS rates of 72%/84% (Figure 1) might have been even better had one patient (#1 in Table 1) received consolidation after achieving CR with induction. This child's relapse, despite a complete pathologic response documented at 2^nd^-look surgery, underscores the importance of consolidative measures.

In past reports on HR-NB [23, 32], we assessed multiple prognostic markers in univariate and multivariate analyses, but in the current study the low number of events precluded the utility of prognostic markers. Nevertheless, it would appear that for this subset of patients there is the same lack of survival advantage with ASCT as seen in our entire cohort of HR-NB patients [23, 32]. Of note, since 1988, our definition of HR-NB has not included *MYCN*-non-amplified localized (including stage 3) disease which we have managed upfront with surgery alone [18].

Four of the long-term survivors received DFMO or vaccine (patients #10-13 in Table 1) after completing antibody treatment. These investigational therapies are of unproven benefit for HR-NB; it cannot be certain whether their use improved outcome. If efficacy is ultimately confirmed with more data in ongoing formal clinical trials, then either or both might be incorporated into the standard of care for HR-NB.

The MSK results with *MYCN*-amplified stage 2/3 are comparable to the 6-year EFS/OS rates of 83%/83% in the series of 12 similar patients whose consolidation included ASCT+RT, though not anti-G_D2_ antibody or isotretinoin, as presented in the only other report focused specifically on this subset of patients [9]. These EFS/OS rates in turn are better than those in other reports with data on *MYCN*-amplified stage 2/3 treated with conventional chemotherapy ± RT, including 5-year EFS/OS 32%/36% (n=22) [4] and 6-year OS 25% (n=20) [9] in early French studies, and 1/12 (8%) patients surviving in the Italian experience [8].

Inferior results compared to the current MSK report and the French ASCT+RT experience [9] were also seen in the only two published studies with data on ASCT in high-risk stage 2 or 3. Thus, the CCG-3891 trial which involved randomizations of ASCT and isotretinoin, but not routine use of local RT, showed 5-year EFS/OS rates of 25%/25% for *MYCN*-amplified stage 3 (n=24); neither ASCT nor isotretinoin had a significant impact on outcome for the entire stage 3 cohort (n=72), which included *MYCN*-non-amplified disease [14]. In that report, the “overall poor prognosis” led the authors to note that “[f]urther studies are warranted to determine if myeloablative consolidation followed by 13-*cis*-RA maintenance therapy statistically significantly improves outcome.” Similarly, ASCT did not improve survival in the INRG cohort of *MYCN*-amplified stage 2 NB (n=39, including nine ASCT), with 5-year EFS/OS of 57%/67% [13].

Reports on more recent large studies of HR-NB do not include patients with *MYCN*-amplified stage 2/3 [20, 39], have few such patients (n=6 [40], n=4 [41]), or do not separate out the results in this subset [11, 16]. Patients underwent ASCT but local RT was routine in only one of those reports [16] and anti-G_D2_ therapy was not used [40–42] or was received by a minority of patients [11, 16, 20]. For the large cohorts of HR-NB patients in those studies, [11, 16, 20, 39–41] the 5-year EFS rates of 24%-to-47% and the 5-year OS rates of 26%-to-60% were inferior to the outcome of the *MYCN*-amplified stage 2/3 patients in our series and in the report using ASCT+RT [9]. One interpretation of these differences is that in the contemporary era of dose-intensive induction plus aggressive consolidation including local RT, *MYCN*-amplified localized NB has a better prognosis than metastatic HR-NB.

The substantial number of non-ASCT patients in the current report and past reports [23, 32] makes the MSK experience unique because ASCT has been a major component of HR-NB treatment programs since 2000 [42]. Hence, the MSK database offers an opportunity not otherwise available to reassess whether ASCT (which is not standard for other extracranial solid tumors [43, 44]) should be routine for HR-NB. Compelling reasons for revisiting this issue include the recent update of the landmark CCG-3891 trial [27] which showed no OS advantage with ASCT [17], and a recent meta-analysis which found that ASCT for HR-NB did not improve OS [45].

An additional consideration supporting a reeval-uation of ASCT for HR-NB is that the only randomized ASCT studies – dating from 1982-1985 [46], 1991-1996 [14, 17, 28], and 1997-2002 [11] – have limited contemporary relevance because of the low dose-intensity of the induction regimens and the absence or irregular use of local RT and anti-G_D2_ antibody. In fact, local control of soft tissue NB is excellent with dose-intensive chemotherapy, resection, and RT [26, 27], and eradication of even histologically-evident chemoresistant NB in BM is reliably achieved with anti-G_D2_ antibodies [47]. Other drawbacks of the randomized studies are that the control arm in one received no therapy [46] and in another received only oral cyclophosphamide [11], and the cytoreduction ranged from single agent (melphalan)^48^ to multiple agents plus total body irradiation, [14, 17, 28] which is no longer routinely used for NB.

The three randomized studies also preceded modern improvements in salvage therapy and in the early detection of recurrent NB [48] – i.e., interventions that could favorably impact OS. In this regard, two non-ASCT patients (#14 and #15 in Table 1) are in continuous 2^nd^ CR and off all therapy 36+ and 95+ months, respectively, from relapse. These welcome outcomes undermine the long-held view that HR-NB relapse is an ultimately lethal event [49]. This view is why EFS of HR-NB patients has been accepted as the most meaningful measure of treatment efficacy. For curability of HR-NB, however, long-term OS may now supersede EFS endpoints as recent developments offer hope that the equivalence between relapse and lethality may no longer hold true. Thus, as in our two patients, close monitoring [48] can detect localized relapses [23], which might be controlled by surgery and/or focal RT, supplemented by systemic therapies that are non-cross-resistant with prior treatments. Examples include chemotherapy regimens [37, 50] and novel agents [33, 51]. Consolidation of 2^nd^ CR in our two patients included retreatment with 3F8/GM-CSF+isotretinoin, as previously reported [38]. They also received a vaccine used with oral β-glucan; this immunotherapy has shown promise in consolidating 2^nd^ CR [34].

In conclusion, a cautious interpretation of the MSK experience is that ASCT may not be warranted when local RT, anti-G_D2_ antibodies, and isotretinoin are used for consolidation after dose-intensive induction chemotherapy. This possibility is supported by a critical review of ASCT for HR-NB reaching back 30 years [45], the loss of long-term survival advantage with ASCT for stage 3 and 4 in a major randomized study [17], and the absence of a benefit with ASCT for localized HR-NB [13, 14]. A definitive confirmation that ASCT does not improve outcome would require a prospective randomized trial. Discontinuing ASCT for HR-NB would be consistent with the general consensus among pediatric oncologists that this highly toxic treatment is no longer recommended for all other extracranial pediatric solid tumors [43, 44].

## MATERIALS AND METHODS

This report covers all *MYCN*-amplified stage 2/3 patients who received induction [24, 25] at MSK during the era of immunotherapy with 3F8/GM-CSF (2000-2015) [23, 32]. Stage and *MYCN* amplification were defined by international criteria [52, 53]. ASCT post-induction was standard through 2003 [16, 54]. Consolidation included local RT (21 Gy) [27] applied between the 1^st^ and 2^nd^ cycles of 3F8/GM-CSF, with 3F8 at 100 mg/m^2^/cycle, as described [23, 32]. Isotretinoin was taken orally (x6 courses, as described^27^) between cycles of 3F8/GM-CSF, beginning after the 2^nd^ cycle [23, 32]. Informed written consents for all treatments were obtained according to institutional review board rules.

Extent-of-disease evaluations included ^123^I-metaiodobenzylguanidine (MIBG) scan and computed tomography or magnetic resonance imaging of chest-abdomen-pelvis every 3 months. BM aspirates and biopsies obtained from bilateral posterior and anterior iliac crests were studied by histology every 3-6 months. Disease status was defined by the International NB Response Criteria [52], modified to incorporate ^123^I-MIBG findings. CR: no evidence of NB, including normal ^123^I-MIBG scan and BM(-) by histology. VGPR, primary mass reduced by ≥90%, and no evidence of distant disease in soft tissue, bones, or BM; and PD: new lesion or >25% increase in an existing lesion.

Quantitative reverse transcription-polymerase chain reaction was used, as described [23], to assess MRD in BM before the initiation of immunotherapy and then after the 2^nd^ cycle of 3F8/GM-CSF.

EFS and OS were estimated using Kaplan-Meier analyses, calculated from diagnosis or from the start of 3F8/GM-CSF. Events were defined as relapse, secondary neoplasm, or death. OS was defined as time to death or last follow-up.

## Abbreviations

HR-NB: high-risk neuroblastoma
ASCT: autologous stem cell transplant
EFS: event free survival
RT: radiation therapy
OS: overall survival
CR/VGPR: complete remission/very good partial remission
MSK: Memorial Sloan Kettering Cancer Center
GM-CSF: granulocyte-macrophage colony-stimulating factor
BM: bone marrow
MRD: minimal residual disease.
